# Naringin–Dextrin Nanocomposite Abates Diethylnitrosamine/Acetylaminofluorene-Induced Lung Carcinogenesis by Modulating Oxidative Stress, Inflammation, Apoptosis, and Cell Proliferation

**DOI:** 10.3390/cancers15205102

**Published:** 2023-10-22

**Authors:** Eman E. Mohamed, Osama M. Ahmed, Khairy M. A. Zoheir, Ahmed A. G. El-Shahawy, Shadi Tamur, Anwar Shams, Jack T. Burcher, Anupam Bishayee, Adel Abdel-Moneim

**Affiliations:** 1 Physiology Division, Faculty of Science, Beni-Suef University, Beni-Suef 62521, Egypt; eman_ezzeldien@yahoo.com (E.E.M.);; 2 Cell Biology Department, Biotechnology Research Institute, National Research Centre, Cairo 12622, Egypt; khma25@gmail.com; 3 Materials Science and Nanotechnology Department, Faculty of Postgraduate Studies for Advanced Sciences, Beni-Suef University, Beni-Suef 62521, Egypt; ahmedelshahawy382@yahoo.com; 4 Departement of Pediatrics, College of Medicine, Taif University, Taif 21944, Saudi Arabia; shaditamur@tu.edu.sa; 5 Departement of Pharmacology, College of Medicine, Taif University, Taif 21944, Saudi Arabia; 6 Centre of Biomedical Sciences Research, Deanship of Scientific Research, Taif University, Taif 21974, Saudi Arabia; 7 High Altitude Research Center, Taif University, Taif 21944, Saudi Arabia; 8 College of Osteopathic Medicine, Lake Erie College of Osteopathic Medicine, Bradenton, FL 34211, USA; jburcher83447@med.lecom.edu (J.T.B.); or abishayee@gmail.com (A.B.)

**Keywords:** diethylnitrosamine, acetylaminofluorene, lung cancer, naringin, naringin–dextrin nanocomposite, anticancer, anti-inflammatory, antioxidant, apoptosis

## Abstract

**Simple Summary:**

Despite advancements in the field, lung malignancies are the primary cause of cancer-attributable morbidity and mortality on a global scale. Recently, therapeutic agents have demonstrated improved efficacy when combined with nanocarriers to improve their delivery, bioavailability, and efficacy. Accordingly, the aim of this study is to evaluate the effects and mechanisms of action of naringin–dextrin nanocomposites (Nar-Dx-NCs) against diethylnitrosamine (DEN)/2-acetylaminofluorene (2AAF)-induced lung carcinogenesis in male Wistar rats. Naringin’s preventive action against DEN/2AAF-induced lung cancer was amplified using Nar-Dx-NCs, which exerted heightened anti-carcinogenic effects by suppressing oxidative stress, inflammation, and cell proliferation and activating apoptosis.

**Abstract:**

Nanotechnology has proven advantageous in numerous scientific applications, one being to enhance the delivery of chemotherapeutic agents. This present study aims to evaluate the mechanisms underlying the chemopreventive action of naringin–dextrin nanocomposites (Nar-Dx-NCs) against diethylnitrosamine (DEN)/2-acetylaminofluorene (2AAF)-induced lung carcinogenesis in male Wistar rats. DEN was administered intraperitoneally (i.p.) (150 mg/kg/week) for two weeks, followed by the oral administration of 2AAF (20 mg/kg) four times a week for three weeks. Rats receiving DEN/2AAF were concurrently treated with naringin or Nar-Dx-NCs orally at a dose of 10 mg/kg every other day for 24 weeks. Naringin and Nar-Dx-NCs treatments prevented the formation of tumorigenic cells within the alveoli of rats exposed to DEN/2AAF. These findings were associated with a significant decrease in lipid peroxidation, upregulation of antioxidant enzyme (glutathione peroxidase and superoxide dismutase) activity, and enhanced glutathione and nuclear factor erythroid 2–related factor 2 expression in the lungs. Naringin and Nar-Dx-NCs exerted anti-inflammatory actions manifested by a decrease in lung protein expression of tumor necrosis factor-α and interleukin-1β and mRNA expression of interleukin-6, interferon-γ, nuclear factor-κB, and inducible nitric oxide synthase, with a concurrent increase in interleukin-10 expression. The anti-inflammatory effect of Nar-Dx-NCs was more potent than naringin. Regarding the effect on apoptosis, both naringin and Nar-Dx-NCs significantly reduced Bcl-2 and increased Bax and P53 expressions. Moreover, naringin or Nar-Dx-NCs induced a significant decrease in the expression of the proliferator marker, Ki-67, and the effect of Nar-Dx-NCs was more marked. In conclusion, Nar-Dx-NCs improved naringin’s preventive action against DEN/2AAF-induced lung cancer and exerted anticarcinogenic effects by suppressing oxidative stress and inflammation and improving apoptotic signal induction and propagation.

## 1. Introduction

Malignancies of the lung are among the most frequently diagnosed types of cancer in both sexes, affecting 11.6% of the population. Due to its aggressive nature, lung cancer has a high mortality rate worldwide, with over 18% of patients ultimately succumbing to cancer-related complications [1,2]. Risk factors include tobacco use, radiation to the thorax, and exposure to secondhand smoke, radon gas, asbestos, and other carcinogens. Diethylnitrosamine (DEN), for example, is frequently used as a carcinogenic agent in in vivo analyses; it has been reported that rodents exposed to DEN develop lung, liver, skin, and gastrointestinal cancers [3,4]. The metabolism of DEN via the metabolic activation of cytochrome P450 (CYP450)-dependent monooxygenase system results in oxidative stress, leading to cytotoxicity, mutagenicity, and carcinogenesis [5,6]. The lungs are perhaps most sensitive to these effects, as DEN has been established to most frequently cause lung cancer compared to other primary tumors [7]. Reactive oxygen species (ROS) produced during the metabolism of DEN may be a major factor in the emergence of cancer. An imbalance between ROS production and antioxidant capacities leads to DNA damage and oxidative stress, ultimately triggering carcinogenesis [8,9]. Another genotoxic carcinogen, 2-acetylaminofluorene (2AAF), forms covalent interactions between metabolic derivatives of 2AAF and DNA to initiate tumorigenesis in many species [10].

Inflammation plays a significant role as an innate broad defense mechanism against injury and generalized infection [11]. During this process, macrophages produce pro-inflammatory cytokines, including interferon-γ (IFN-γ), tumor necrosis factor-alpha (TNF-α), interleukin-6 (IL-6), as well as other inflammatory proteins, including inducible nitric oxide synthase (iNOS) [12]. Cytokines are essential regulators of the immune and inflammatory responses via intricate networks and, therefore, serve as biomarkers for disease [13]. Key pro-inflammatory cytokines implicated in tumorigenesis include TNF-α, interleukin-1β (IL-1β), and IL-6 [14,15]. Tumorigenesis and inflammation are closely related, and much effort pertaining to cancer research is focused on this topic [16]. For example, the crucial importance of nuclear factor erythroid 2-related factor 2 (Nrf2) was recently shown to control the antioxidant response element (ARE), which protects against oxidative stress [17].

Natural sources of antioxidants are an increasingly attractive option for chemoprevention due to their relatively nontoxic side effect profiles [18]. Naringin, a polyphenolic phytochemical belonging to the flavonoid subclass, has already been reported to possess antioxidant, anti-inflammatory, and antitumor effects [19,20]. Targeted drug delivery systems have been substantially advanced in recent years, allowing drugs to be delivered to specific locations to treat a variety of diseases with greater efficacy and, in theory, reduced toxicity [21]. Compared to native formulations, the solubility, release profiles, and bioavailability of nanoscale drugs, in conjunction with their nano-based delivery systems, have proven beneficial for cancer treatment. The use of targeted nano-based drug delivery systems permits a more appropriate route of administration, fewer adverse effects, diminished toxicity, greater diffusivity, and a longer half-life of anticancer drugs [22]. Previous research has demonstrated that nanomaterials can selectively enter organs, tissues, and cells to release treatments at precise locations previously considered to be inaccessible without great difficulty [23,24]. Combining nanodrug formulations with natural products may further reduce drug toxicity and augment the therapeutic properties of both conventional agents and natural compounds [25,26,27,28,29]. Newly published in vitro and in vivo data are already in support of naringin–dextrin nanocomposites (Nar-Dx-NCs) exhibiting anti-inflammatory and anticancer effects against hepatocellular carcinoma, but information regarding the effects of Nar-Dx-NCs in the context of lung cancer is lacking [30,31]. Therefore, the purpose of our study is to report the anticarcinogenic effects and mechanisms of action of Nar-Dx-NCs in DEN/2AAF-induced lung carcinogenesis in male Wistar rats.

## 2. Materials and Methods

### 2.1. Chemicals

DEN (Cat. #049K1613V), 2AAF (Cat. #A7015), naringin (Cat. #BCBT3477), and dextrin (Cat. # D2006) were obtained from Sigma-Aldrich (St. Louis, MO, USA). All other chemicals and solvents utilized were of analytical grade.

### 2.2. Synthesis of Nar-Dx-NCs

Nar-Dx-NCs were prepared according to the method described by Manchu et al. [32]. Briefly, a 5% (*w*/*w* or 0.2 mg/mL) aqueous nanoemulsion of dextrin and naringin was produced by continuous ultrasonication for 1 min. Formaldehyde was then added in exact molar ratios to dextrin, homogenized via ultrasonication at high pressure for 30 min, and stirred at room temperature for 12 h. After precipitating with ethanol 99% (*v*/*v*), the crosslinked dextrin–naringin nanocomposite was centrifuged at 14,000 rpm for 45 min at 4 °C before being frozen for 48 h. The nanoemulsion was stored as a powder at 4 °C. In our earlier publication [30], we outlined the techniques utilized to characterize the Nar-Dx-NCs, including X-ray diffraction (XRD), Fourier transformation infrared (FTIR) spectroscopy, transmission electron microscopy (TEM), zetasizer and zeta potential studies, and ultraviolet-visible (UV-Vis) spectrometry. Additionally, Nar-Dx-NCs were evaluated according to entrapment effectiveness (EE) and the release of naringin from dextrin. 

### 2.3. Animal Experimentation

Wistar male adult rats weighing 100–120 g and aged 7–8 weeks were utilized in this study. The animals were purchased from the Animal House Facility of the Egyptian Organization for Biological Products and Vaccines (VACSERA, Cairo, Egypt). Rats were observed for 7 days prior to the start of experimental procedures to allow acclimation to their new environment and monitor for infection. The rats were kept in a controlled environment with a 12/12 h light/dark cycle, a standard diet, and unrestricted access to water. The temperature was kept between 20 and 25 °C. All procedures involving animals conducted in this present study were approved by the Animal Ethics Committee of the Faculty of Science at Beni-Suef University in Egypt (Approval Number: BNS:020-91). 

### 2.4. Experimental Design

**Figure 1:**
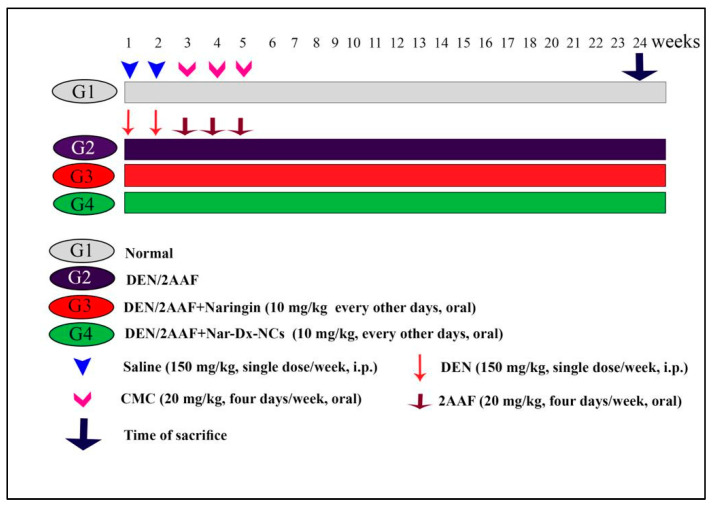
Schematic of the experimental design. Abbreviations: 2AAF, 2-acetylaminofluorene; CMC, carboxymethyl cellulose; DEN, diethylnitrosamine; Nar-Dx-NCs, naringin–dextrin nanocomposites.

Adult male Wistar rats were divided into four groups, each comprising 12 rats. Group 1 served as an untreated normal group, while groups 2, 3, and 4 received 150 mg/kg of DEN intraperitoneally (i.p.) twice a week for two weeks plus 20 mg/kg 2AAF via oral gavage four times a week for three weeks [33,34]. Group 2 served as the positive control group and received no further interventions. Groups 3 and 4 received naringin or Nar-Dx-NCs, respectively, at a dose of 10 mg/kg every other day from the start of carcinogenesis for the duration of this study (24 weeks), according to a previously established protocol [35]. Groups 1 and 2 received the same volume of the vehicle (5 mL 1% carboxymethyl cellulose), in which naringin and Nar-Dx-NCs were dissolved. After 24 weeks, the animals were anesthetized with diethyl ether before being sacrificed (Figure 1).

### 2.5. Lung Sampling and Analysis

**Figure 2:**
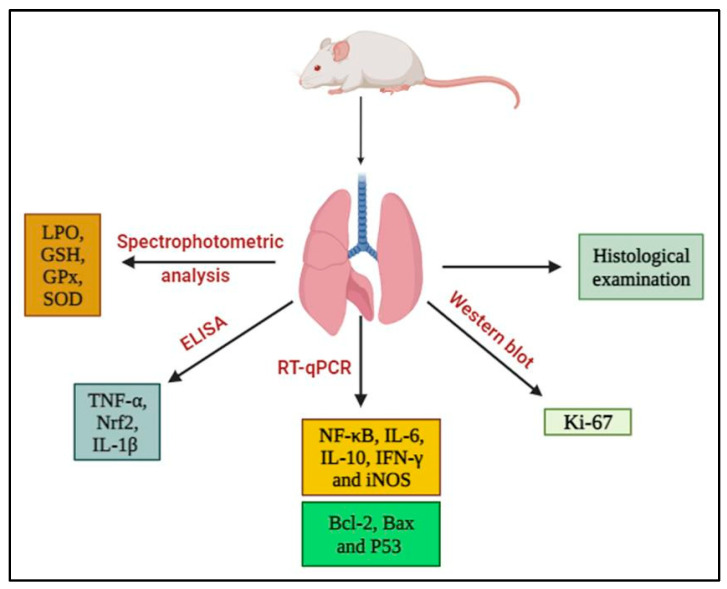
Schematic diagram of analyses performed on lung samples and endpoints recorded. Abbreviations: Bax, Bcl-2-associated X protein; Bcl-2, B-cell lymphoma-2; ELISA, enzyme-linked immunosorbent assay; GSH, glutathione; GPx, glutathione peroxidase; Ki-67, Kiel-67; IFN-γ, interferon-γ; IL-1β, interleukin-1β; IL-6, interleukin-6; IL-10, interleukin-10; iNOS, inducible nitric oxide synthase; LPO, lipid peroxidation; NF-κB, nuclear factor-κB; Nrf2, nuclear factor erythroid 2-related factor 2; RT-qPCR, reverse transcription–quantitative polymerase chain reaction; SOD, superoxide dismutase.

The lung parenchyma of euthanized rats was resected and washed with cold sterile saline. Each lung was cut into four pieces (3 mm^3^). After one day of fixation in 10% neutral buffered formalin, one lung piece was cut into sections for histological analysis. Another lung sample was mixed with phosphate buffer at 25% *w*/*v* and centrifuged at 3000 rpm for 15 min at 4 °C. The supernatants were stored at −30 °C until they were used to investigate antioxidant activities, oxidative stress, and biomarker levels, including TNF-α, IL-1β, and Nrf2. The third portion of the lung tissue was kept at −70 °C in sterilized tubes and used for RNA isolation and real-time polymerase chain reaction (PCR) assays. A fourth lung tissue sample was kept at −30 °C and was used for Western blot analysis of proliferator marker, Kiel-67 (Ki-67). Various experimental endpoints are illustrated in Figure 2.

### 2.6. Histopathological Examination

Additional samples of lung tissue fixed for 24 h in 10% neutral buffered formalin were dehydrated with ethyl alcohol and embedded in paraffin. The lung sections (5 mm^3^) were then stained with hematoxylin and eosin (H&E) according to the method described by Bancroft and Gamble [36] and examined under a light microscope. The histopathological lesions of lung sections stained with H&E were evaluated via histomorphometric analysis using ImageJ 1.46r software (Leica Qwin 500 image system, Cambridge, UK) [37]. The mean area of alveoli space and mean area percentages of inflammation and hemorrhage were measured. 

### 2.7. Biochemical Assays

Lung lipid peroxide (LPO), reduced glutathione (GSH), glutathione peroxidase (GPx), and superoxide dismutase (SOD) were analyzed using kits from Bio-Diagnostic (Dokki, Giza, Egypt) according to the manufacturer’s instructions. Antibodies against TNF-α, IL-1β, and Nrf2 were used on the lung tissue obtained from rats using Enzyme-Linked Immunosorbent Assay (ELISA) kits following the guidelines of the manufacturer (R&D Systems, Minneapolis, MN, USA). 

### 2.8. Isolation of Total RNA and Reverse Transcription–Quantitative PCR (RT-qPCR)

Total RNA was extracted from homogenates of lung tissue using the TRIzol reagent (Cat. # 15596018, Invitrogen; Eugene, OR, USA) according to the manufacturer’s instructions. RNA absorbance was measured at 260 nm using the method described by Sthoeger et al. [38]. The RNA quality was ascertained by measuring the ratio of the absorbance at 260 and 280 nm (260/280 ratio). The first strand of cDNA was synthesized using the high-capacity cDNA reverse transcription kit (Cat. # 4368814) from Applied Biosystems^®^ (Foster City, CA, USA). Quantitative analyses of the target genes’ mRNA expression were performed via quantitative PCR using the ABI Prism 7500 System (Biosystems, Foster City, CA, USA) and 96-well optical reaction plates. In brief, the cDNA was amplified via PCR (Applied Biosystems, Foster City, CA, USA) using a 25-μL reaction mixture, including 0.1 μL of 10 μM forward primer and 0.1 μL of 10 μM reverse primer each (final concentration of each primer: 40 nM), 11.05 μL of nuclease-free water, 12.5 μL of SYBR Green Universal Master mix, and 1.25 μL of cDNA sample. Table 1 contains a list of the primers used in this present study. The primers were obtained from Santa Cruz Biotechnology, Inc., Heidelberg, Germany. Results are displayed as a fold change in the gene expression normalized to that of the reference gene β-actin using the 2^−ΔΔCT^ method.

### 2.9. Western Blot Analysis

Lung tissues were homogenized in radioimmunoprecipitation assay (RIPA) buffer and centrifuged to obtain clear supernatants. The total protein content was measured using the Bradford reagent (SK3041) (Bio Basic Inc., Markham, ON, Canada). Afterward, 30 µg of proteins were separated using sodium dodecyl sulfate–polyacrylamide gel electrophoresis (SDS–PAGE) (Cat. # 161-0181; Bio-Rad Laboratories, Inc., Hercules, CA, USA) and then transferred to nitrocellulose membranes. After blocking in tris-buffered saline with Tween 20 (TBST) containing 5% non-fat milk powder, the membranes were incubated with primary antibodies against Ki-67 (Cat. # AB9260; EMD Millipore Corporation, Temecula, CA, USA) and β-actin (Sc-8432). The membranes were developed with the help of the chemiluminescent substrate (Cat. # 170-5060) (ClarityTM Western ECL substrate, Bio-Rad Laboratories, Inc., Hercules, CA, USA) and then examined with the corresponding horseradish peroxidase (HRP)-conjugated secondary antibodies (goat anti-rabbit IgG-HRP-1 mg goat monoclonal antibody) (Novus Biologicals, Littleton, CO, USA). Finally, the intensity ratio in the control sample was compared with the band intensities of the target proteins using image analysis (ChemiDoc MP Imaging system, Bio-Rad, Hercules, CA, USA) normalized to that of the housekeeping protein β-actin.

### 2.10. Statistical Analysis

The data are shown as means ± standard errors (SE). A one-way analysis of variance was performed using SPSS version 20 software (Chicago, IL, USA) to identify statistical differences between groups. Duncan’s test was then used to compare various groups, with significance set at *p* < 0.05.

## 3. Results

### 3.1. Naringin and Nar-Dx-NCs Abrogated Histopathological Changes in Lung Parenchyma of Rats Administered DEN/2AAF

**Figure 3:**
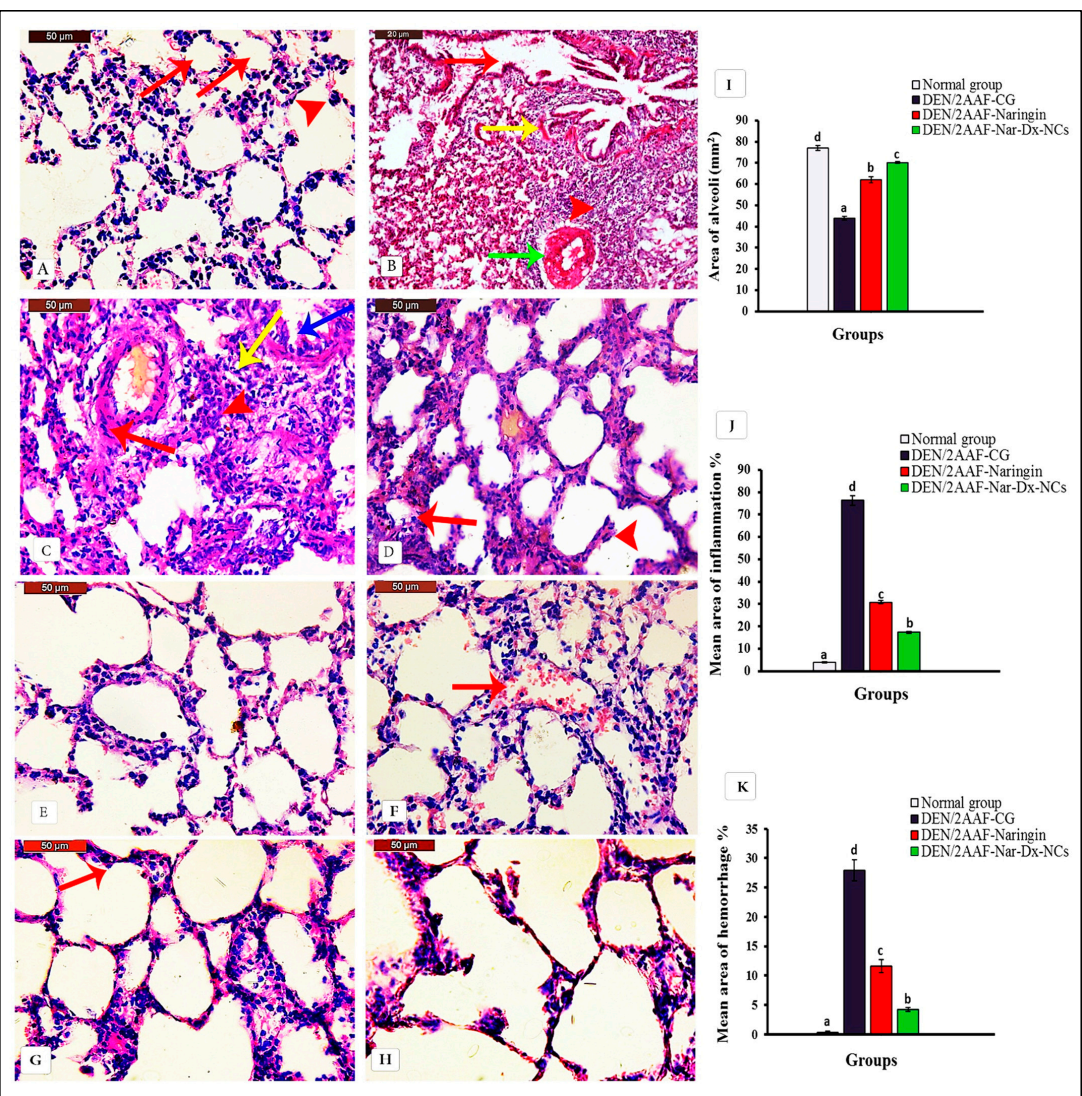
(**A**) Photomicrographs of sections of the lungs from control rats showing normal histology of the alveolar area, including thin and delicate walls (red arrow). The alveoli were well aerated and contained only an occasional pulmonary macrophage (red arrowhead) (hematoxylin and eosin [H&E] staining, scale bar: 50 µm). (**B**) Photomicrograph of lung section from DEN/2AAF-administered rat showing lung cancerous injuries including aggregation of tumor cells (red arrowhead) diffuse thickening in interstitial tissues (red arrow), hemorrhage (yellow arrow), and congested blood vessel (green arrow) (H&E staining, scale bar: 20 µm). (**C**) Photomicrograph of lung section from DEN/2AAF-administered rat displays invasive adenocarcinoma with an acinar pattern. Tumor is seen invading into a vessel (red arrow). Typical lepidic (blue arrow) and papillary (yellow arrow) growth patterns of tumor cells within alveoli. Macrophages and other myeloid cells infiltrated adjacent alveolar air spaces (red arrowhead) (H&E staining, scale bar: 50 µm). (**D**) Photomicrograph of lung section from DEN/2AAF-administered rat showing lepidic (red arrow) and papillary (red arrowhead) growth patterns of tumor cells within alveoli. Macrophages and other myeloid cells infiltrated adjacent alveolar air spaces (red arrow) (H&E staining, scale bar: 50 µm). (**E**,**F**) Photomicrographs of lung sections of DEN/2AAF-administered rats treated with naringin showing a relatively normal structure of the alveolar walls. A hemorrhage was found in the lumen of some alveoli (red arrow) (H&E staining, scale bar: 50 µm). (**G**,**H**) Photomicrographs of lung sections of DEN/2AAF-administered rats treated with Nar-Dx-NCs showing a near-normal structure of the alveolar walls. Occasional pulmonary macrophages (arrow) were observed (H&E staining, scale bar: 50 µm). (**I**–**K**) Graphs displaying the mean area of alveoli to determine tumor cells within alveoli and mean area percentages of inflammation and hemorrhage, respectively, in each group studied. Means for each group are represented at the top of each bar. Different symbols (a–d) within the same graph are significantly different at *p* < 0.05. Abbreviations: 2AAF, 2-acetylaminofluorene; CG, control group; DEN, diethylnitrosamine; Nar-Dx-NCs, naringin–dextrin nanocomposites.

The histopathological examination of lung sections from the normal group revealed normal histology with thin and delicate walls in the alveolar area. The alveoli were well-aerated and contained only occasional pulmonary macrophages (Figure 3A). In contrast, rats receiving DEN/2AAF presented histological abnormalities consistent with carcinogenesis, including aggregation of tumor cells and diffuse thickening in interstitial tissues, with hemorrhage and congested blood vessels (Figure 3B). Tumor cells grew within the alveoli following lepidic and papillary growth patterns. Additionally, macrophages and other myeloid cells invaded adjacent alveolar air spaces. Tumor cells also displayed an acinar pattern of invasive adenocarcinoma. The invasion of a vessel via tumor cells was also observed (Figure 3C,D).

The structure of the alveolar walls of the lungs of DEN/2AAF-administered rats treated with naringin was relatively similar to that of healthy controls (Figure 3E). A hemorrhage was found in the lumen of some alveoli (Figure 3F). The structure of the alveolar walls of the lungs of DEN/2AAF-administered rats treated with Nar-Dx-NCs also appeared nearly normal. Occasional pulmonary macrophages were observed (Figure 3G,H). The statistical analysis of the mean area of alveoli space to determine tumor cells within alveoli, mean area percentages of inflammation, and hemorrhage confirmed an improvement in DEN/2AAF-administered groups treated with native or nanocomposite naringin compared to those not receiving concurrent chemoprophylaxis. These findings were more pronounced in Group IV, which received Nar-Dx-NCs, compared to native naringin (Group III) (Figure 3I–K). 

### 3.2. Effect of Naringin and Nar-Dx-NCs on Oxidative Stress and Antioxidant Defense System in Lung Tissues

DEN/2AAF administration significantly (*p* < 0.05) elevated lung LPO and reduced the lung GSH content as well as GPx and SOD activities compared with those of normal rats. Free naringin or Nar-Dx-NCs treatment significantly (*p* < 0.05) reduced lung LPO and could increase GSH content and GPx and SOD activities compared with the values obtained in rats receiving DEN/2AAF alone (Table 2). 

### 3.3. Effects of Naringin and Nar-Dx-NCs on TNF-α, IL-1β, and Nrf2 Levels in Lung Tissues

**Figure 4:**
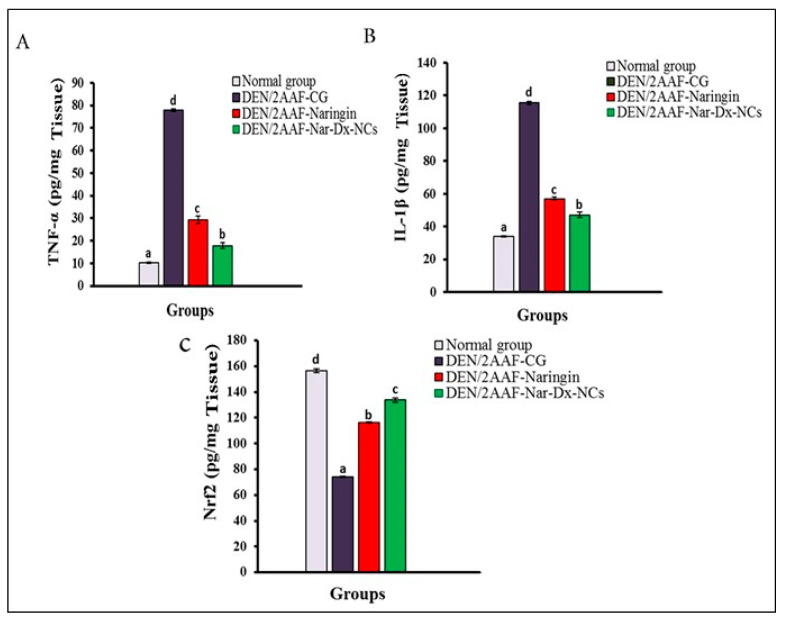
Effect of naringin or Nar-Dx-NCs on (**A**) TNF-α, (**B**) IL-1β, and (**C**) Nrf2 levels in the lungs of DEN/2AAF-administered rats. Data are expressed as means ± SE (*n* = 6). Means for each group are represented at the top of each bar. Different symbols (a–d) within the same graph are significantly different at *p* < 0.05. Abbreviations: 2AAF, 2-acetylaminofluorene; CG, control group; DEN, diethylnitrosamine; IL-1β, interleukin-1β; Nar-Dx-NCs, naringin–dextrin nanocomposites; Nrf2, nuclear factor erythroid 2-related factor 2; TNF-α, tumor necrosis factor-α.

TNF-α and IL-1β levels in lung tissues were higher in DEN/2AAF-administered rats, whereas Nrf2 levels were lower (*p* < 0.05) compared to normal rats. In DEN/2AAF-administered rats, treatment with naringin or Nar-Dx-NCs reversed this effect (Figure 4).

### 3.4. Effects of Naringin and Nar-Dx-NCs on IL-6, IL-10, NF-κB, IFN-γ, and iNOS mRNA Expression in Lung Tissues 

**Figure 5:**
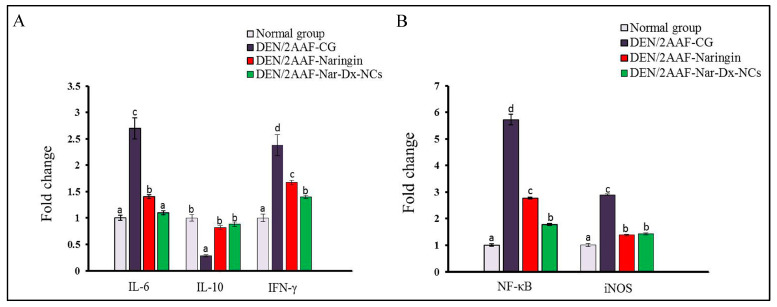
Effect of naringin and Nar-Dx-NCs on (**A**) IL-6, IL-10, and IFN-γ mRNA expressions as well as (**B**) NF-κB and iNOS mRNA levels in the lung of DEN2AAF-administered rats. Data are expressed as means ± SE (*n* = 6). Means for each group are represented at the top of each bar. Different symbols (a–d) within the same graph are significantly different at *p* < 0.05. Abbreviations: IFN-γ, interferon-γ; IL-10, interleukin-10; IL-6, interleukin-6; iNOS, inducible nitric oxide synthase; NF-κB, nuclear factor-κB.

DEN/2AAF-administered rats exhibited greater IL-6, NF-κB, IFN-γ, and iNOS levels in lung tissues and a significant decrease in IL-10 expression compared with normal rats. The treatment of DEN/2AAF-administered rats with free naringin or Nar-Dx-NCs had a significant effect on the increase in IL-6, NF-κB, IFN-γ, and iNOS mRNA expressions and decreased IL-10 mRNA levels compared with those in DEN/2AAF rats (Figure 5).

### 3.5. Effects of Naringin and Nar-Dx-NCs on mRNA Expression of Antiapoptotic and Proapoptotic Biomarkers 

**Figure 6:**
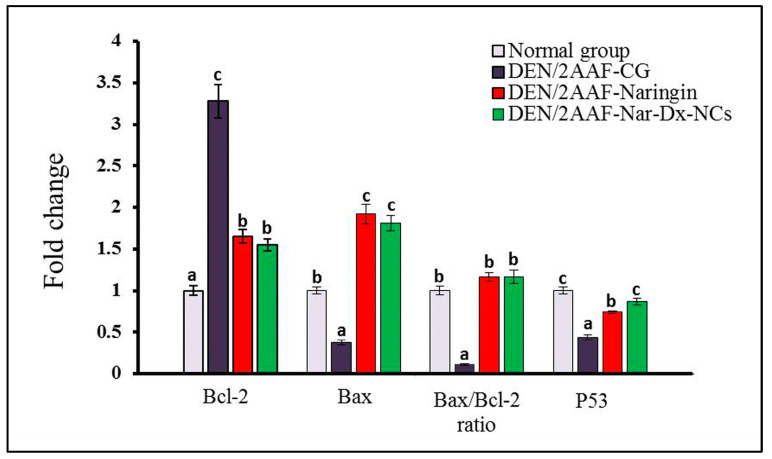
Effect of naringin and Nar-Dx-NCs on Bcl-2 and Bax mRNA levels, Bax/Bcl-2 ratio, and P53 mRNA levels in the lungs of DEN/2AAF-administered rats. Data are expressed as means ± SE (*n* = 6). Means for each group are represented at the top of each bar. Different symbols (a–c) within the same graph are significantly different at *p* < 0.05. Abbreviations: 2AAF, 2-acetylaminofluorene; Bax, Bcl-2-associated X protein; Bcl-2, B-cell lymphoma-2; CG, control group; DEN, diethylnitrosamine; Nar-Dx-NCs, naringin–dextrin nanocomposites.

DEN/2AAF-administered rats produced a significant elevation in the lung expression of antiapoptotic protein B-cell lymphoma-2 (Bcl-2) and significantly reduced levels of the proapoptotic factors Bcl-2-associated X protein (Bax) and P53 compared with those in normal rats. The cotreatment of DENA/2AAF plus free naringin or Nar-Dx-NCs significantly suppressed Bcl-2 expression and elevated Bax and P53 levels (Figure 6).

### 3.6. Effects of Naringin and Nar-Dx-NCs on Ki-67 Protein Expression 

**Figure 7:**
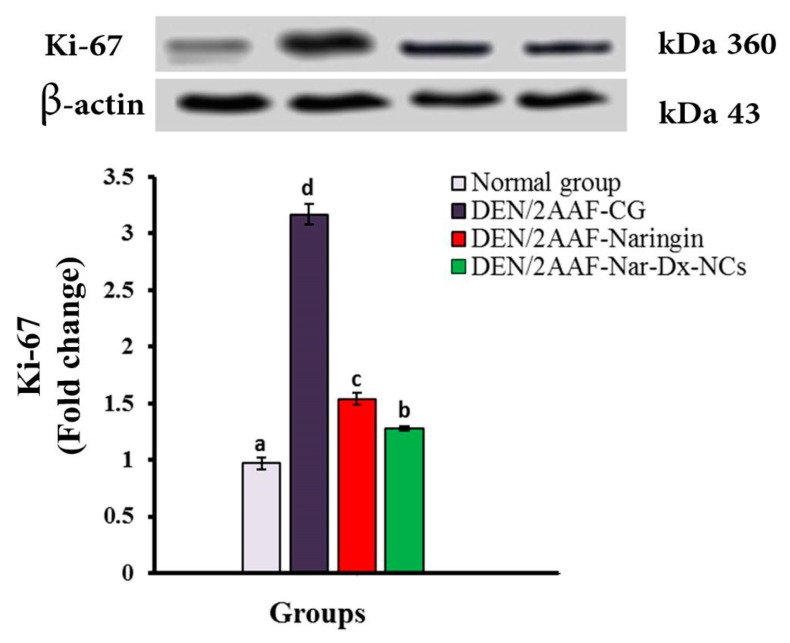
Effect of naringin and Nar-Dx-NCs on Ki-67 levels in the lungs of DEN/2AAF-administrated rats. Data are expressed as means ± SE (*n* = 3). Means, which are represented as different symbols (a–d) at the top of each bar, were considered significantly different for *p* < 0.05. Abbreviations: 2AAF, 2-acetylaminofluorene; Bax, Bcl-2-associated X protein; Bcl-2, B-cell lymphoma-2; CG, control group; DEN, diethylnitrosamine; Ki-67, Kiel-67; Nar-Dx-NCs, naringin–dextrin nanocomposites. The uncropped blots are shown in Figure S1 and S2.

Ki-67 protein expression was significantly (*p* < 0.05) upregulated in the lung parenchyma of DEN/2AAF-administered rats. Naringin or Nar-Dx-NCs significantly downregulated lung Ki-67 expression levels compared with those measured after DEN/2AAF administration alone, suggesting an inhibition of cell proliferation (Figure 7).

## 4. Discussion

Lung cancer is a common neoplasm with high incidence and mortality worldwide [2]. DEN is a chemical compound widely utilized to cause lung, liver, and esophageal cancers in experimental animals [39] and may be used alone or in conjunction with a cancer promoter, such as 2AAF [34]. Naringin is the primary bioactive polyphenol found in citrus fruits, and its consumption has been beneficial to human health since ancient times. Numerous preclinical and clinical findings have documented its antioxidant activity and capacity to protect against inflammation and various organ- or system-specific cancers [40]. In this work, we have prepared naringin–dextrin nanocomposites (Nar-Dx-NCs) and investigated the chemopreventive role of this nanoformulation against DEN/2AAF-induced lung cancer and related mechanisms of action compared to free naringin in a Wistar rat model.

The histopathological analysis revealed various lung cancerous injuries, including aggregation of tumor cells, diffuse thickening in interstitial tissues, and tumor cell growth in lepidic and papillary patterns within the alveoli in the lungs of rats given DEN/2AAF. Moreover, there was an invasion of macrophages and other myeloid cells in adjacent alveolar air spaces and a display of invasive adenocarcinoma with an acinar pattern. Based on these observations, it has been demonstrated that lung adenocarcinoma is predominantly caused by DEN, as described by Sivalingam et al. [39] and Abdel-Moneim et al. [34]. This present study revealed an improvement in tissue dysplasia and several biomarkers resulting from cotreatment with naringin or Nar-Dx-NCs, which can minimize the production of ROS induced via DEN injection, thus mitigating tissue damage and cancer-related tissue injury. Compared to free naringin, Nar-Dx-NCs were more effective in exerting anticarcinogenic effects.

DEN treatment increased ROS and LPO levels and decreased SOD, GPx, and GSH activities. These effects were similar to those of Sivalingam et al. [39] and Abdel-Moneim et al. [34], who indicated that DEN increased LPO levels and decreased GPx and SOD activities as well as GSH content in the lungs of rats exposed to DEN. Enzymatic and non-enzymatic antioxidants, like GPx, SOD, and GSH, scavenge ROS and decrease LPO levels. Many studies [31,34,41] found a significant decrease in SOD, GPx, and GSH activities in rat lung and liver cancers, along with increased free radicals and specific humoral factors. LPO levels increase during the development of cancer and are regarded as a strong carcinogen and mutagen [42]. Naringin or Nar-Dx-NCs treatment reduced high LPO levels and increased SOD and GPx activities and GSH content in the lungs. Our data are parallel with the findings of Kim et al. [43]. Naringin, a naturally occurring flavonoid, displays protective effects and increases the activity of antioxidant enzymes in the lungs [44]. The antioxidant defense system in the rat was improved using Nar-Dx-NCs [30]. Dietary antioxidants may help to reduce oxidative stress and cellular damage in addition to supporting the body’s natural antioxidant defense system. There is proof that naringin scavenges free radicals and prevents oxidative DNA damage [45]. Nar-Dx-NCs improved the antioxidant defense system in the rat lungs compared with the effects of crude naringin at the same dose, mainly by increasing naringin concentration and enhancing naringin protective activity. 

Inflammation is the primary response to infections and injuries, but tissue damage may result from an uncontrolled prolongation of the inflammatory repertoire [46]. Recent studies have focused on the cytokines involved in the induction and maintenance of inflammation. TNF-α, IL-1β, IL-6, and INF-γ, as well as the anti-inflammatory IL-10, are the chief mediators of inflammation [47,48]. Our results showed an elevation in TNF-α, IL-1β, NF-κB, and IL-6 expressions, in addition to decreased Nrf2 and IL-10 levels, in lung tissues of DEN/AAF2-administered rats compared to unexposed controls. These results corroborate the findings of previous studies by Man et al. [49], Wu et al. [50], and Cicek et al. [51]. In this current study, the amounts of TNF-α, IL-1β, and IL-6 increased after treating rats with DEN/2AAF but were abrogated using both free naringin and Nar-Dx-NCs [52,53,54]. The DEN/2AAF-induced production of ROS, which activates oxidative stress, may be a contributing factor to the chronic inflammation developing in lung tissues. Lung cancer is also caused by chronic inflammation, and many pro-inflammatory genes, including TNF-α and IL-6, are crucial for apoptosis, proliferation, angiogenesis, and metastasis inhibition [55]. Importantly, the present data indicated that the Nar-Dx-NC formula was significantly more potent than free naringin in mediating this inflammatory response.

Nrf2 is essential for lung cancer cell protection because it is responsible for compensatory mechanisms responsible for attenuating oxidative stress (Figure 6). Additionally, it also controls the inflammatory response within the tumor microenvironment by reducing the expression levels of TNF-α, IL-1β, and IL-6 [56]. Nrf2 is inhibited in malignant lung tumors [57]. IL-10 facilitates immunosuppressive actions and is produced in a variety of solid tumors and hematopoietic tumors. Moreover, it inhibits the synthesis of a number of cytokines, including different inflammatory and growth factors, and promotes the release of anti-inflammatory factors in the body [58]. The levels of IL-10 were effectively restored to near-normal levels via treatment with free naringin or Nar-Dx-NC. In this present study, rats treated with DEN/2AAF also showed a significant elevation in IFN-γ expression compared to rats receiving no interventions. IFN-γ plays a significant role in the contribution of inflammation to carcinogenesis [59], and there is a long history of IFN-γ use as a pro-tumor factor [60]. Chronic uncontrolled inflammation continuously generates harmful ROS, which can change the genome and cause DNA damage, consequently initiating the growth of tumors. Inflammatory mediators such as TNF-α, IL-1β, IL-6, and IFN-γ promote an increase in blood supply to the tumor [61,62].

NF-κB is a transcription factor that regulates the cell cycle, inflammation, and cell survival. NF-κB expression was found to be significantly increased in DEN/2AAF-induced animals relative to that of the control group. These results agree with those of Hamza et al. [63], who suggested that, under oxidative stress, NF-κB becomes activated and translocates to the nucleus to activate inflammatory genes, ultimately facilitating an oncogenic environment. Inflammation has been shown to be stimulated via the upregulation of inflammatory-related genes, such as NF-κB [64]. The pro-inflammatory mediators are primarily controlled via NF-κB, which is induced via chemical carcinogens and is constitutively active in most tumors [65]. When NF-κB is activated, the expression of many genes, including those involved in the production of pro-inflammatory cytokines TNF-α, IFN-γ, IL-1β, and IL-6, is upregulated [66,67]. DEN/2AAF significantly increased these pro-inflammatory cytokines, whereas naringin and Nar-Dx-NCs suppressed this phenomenon. The NF-κB pathway is essential for the survival of cancer cells and their resistance to apoptosis. Naringin inhibits the growth, promotion, and spread of cancer via a variety of mechanisms, including the modulation of numerous uncontrolled signaling pathways related to inflammation, apoptosis, proliferation, angiogenesis, autophagy, invasion, and metastasis [68].

In this present study, iNOS gene expression significantly increased in lung tissue samples of rats administered DEN/2AAF compared to unexposed controls. This finding is supported by the conclusions of Unsal and Kurutaş [69], who found that DEN cytotoxicity and carcinogenicity were attributed to their ability to cause oxidative stress and toxicity. During the inflammatory response, macrophages produce a large amount of nitric oxide (NO), an inflammatory mediator, thanks to the catalytic activity of iNOS. Moreover, excessive NO can cause severe oxidative stress and exacerbate tissue injury [70]. When iNOS is activated via DEN, endogenous NO is produced and causes damage and inflammation. Superoxide anion (O2) and NO interact non-enzymatically to form the reactive nitrogen species peroxynitrite (ONOO^−^). ONOO^−^ causes protein oxidation when it interacts with amino acids. Additionally, ONOO^−^ oxidizes nuclear tyrosine, DNA, and other aromatic amino acids. The nitration of tyrosine produces 3-nitrotyrosine (3-NT), which is a biomarker of oxidative damage and inflammation [71]. The expression of iNOS is affected by endogenous pro-inflammatory mediators like IFN-γ, TNF-α, IL-1β, and IL-6, and iNOS induced via inflammatory stimuli contributes to a large amount of NO production [72].

Treatment with naringin and Nar-Dx-NCs might protect rats from DEN/2AAF-induced inflammation via the downregulation of pro-inflammatory cytokines, such as TNF-α, IL-1β, IL-6, and INF-γ, and by increasing IL-10 and Nrf2 levels compared to those of rats treated with DEN/2AAF alone. Naringin treatment may minimize tissue damage by lowering pro-inflammatory biomarkers levels and stimulating anti-inflammatory mediator production in lung tissue [73]. Naringin reduces inflammation in the rat lungs by decreasing iNOS and TNF-α levels via NF-κB signaling inhibition [74]. Additionally, evidence suggests that naringin is effective at reducing the expression of a number of signaling molecules involved in the inflammatory response other than NF-κB, iNOS, and TNF-α, including IL-6 and Nrf2 [75]. The levels of pro-inflammatory mediators were decreased after treatment with naringin and Nar-Dx-NCs. Thus, naringin and Nar-Dx-NCs treatments inhibit the release of cytokines and are extremely effective against DEN/2AAF-induced lung cancer. The mechanisms triggered by naringin for its anti-inflammatory action might include the inhibition of iNOS and NF-κB signaling pathways [76,77].

Apoptosis results from the activation of the intrinsic and/or extrinsic pathways. The tumor suppressor p53 is a powerful apoptosis regulator that controls the expression of apoptosis-related genes, such as Bax and Bcl-2 [78,79]. Our data showed that exposure to DEN/2AAF elevated Bcl-2 expression while reducing Bax and P53 expression in the lung tissues of Wistar rats. The upregulation of lung Bcl-2 and the reduction in Bax expression in DEN/2AAF-administered animals agrees with the observations of Sivalingam et al. [39]. Campbell and Tait [80] previously demonstrated that the Bcl-2 family, when unbalanced, can act as a barrier to apoptosis and facilitate tumor development and resistance to cancer therapy. The antiapoptotic Bcl-2 negatively regulates apoptosis by inhibiting the activity of the proapoptotic Bax [81]. Bax, Bcl-2, and cytochrome *c* control the apoptotic pathway, which culminates in the activation of caspases [82]. The Bax/Bcl-2 ratio regulates the activation of caspase [83] and thus controls the cell’s decision toward survival or apoptosis.

The present data showed that when DEN/2AAF was administered, lung P53 expression was significantly reduced compared to that of unexposed controls. This finding is supported by the work of Abdel-Moneim et al. [34]. Two functions of P53 are involved in the regulation of oxidative stress; it has prooxidant activity that causes oxidative damage, but it also acts as an antioxidant factor to prevent oxidative stress. Thus, numerous variables, including the absence of a functional P53, contribute to the overproduction of ROS [84]. Human tumors with genetic mutations were found to have inactivated p53, which contributes to the development of tumors from normal cells [85]. Here, treatment with naringin or Nar-Dx-NCs enhanced apoptosis in lung cancer. On the other hand, elevated levels of Bax and P53 and reduced Bcl-2 expression were detected in the lung tissue of DEN/2AAF-administered animals receiving naringin or Nar-Dx-NCs. Naringin has previously been shown to inhibit tumor cell growth, promote apoptosis of tumor cells, and have great potential as a cytotoxic anticancer agent [86,87]. Flavonoids like naringin affecting oxidative stress and apoptosis were shown to stimulate Bax and P53 expression and inhibit Bcl-2 expression in past works [40,88]

Ki-67 is a nuclear marker associated with tumor cell proliferation that has been linked to the progression, metastasis, and prognosis of many cancers [89]. Numerous studies have suggested that patients harboring lung adenocarcinoma with high tumor Ki-67 expression have a worse prognosis, and Ki-67 expression may be an important prognostic factor in advanced lung cancer cases [90,91]. The present findings demonstrate a significant increase in Ki-67 expression levels in the lungs of rats exposed to DEN/2AAF, a finding that has also been reported by Abdel-Moneim et al. [34]. DEN/2AAF-administered rats treated with naringin and Na-Dx-NCs expressed less Ki-67 than DEN/2AAF-administered rats. Naringin is a prooxidant and may inhibit the growth of cancer cells by blocking NF-κB signaling [40]. Thus, the anticancer effects of Nar-Dx-NCs might involve both the inhibition of cancerous cell proliferation and stimulation of apoptosis [30]. Nar-Dx-NCs were significantly more potent than free naringin. Additional efforts are required to clarify the mechanisms involved in the preventive effect of Nar-Dx-NCs on lung cancer and identify other related factors.

The prolonged-release properties of naringin may be responsible for Nar-Dx-NCs potent anticancer properties, indicating dextrin’s consistency as an effective nanocarrier for long-term naringin delivery [30]. The nanocarriers enable a controlled (sustained) drug release from the matrix. Enhanced transport to, or uptake by, target cells may be another reason the nanocomposite impact is superior in addition to sustained release [92]. Nar-Dx-NCs is more potent than free naringin against oxidative stress, inflammation, and cell proliferation may be due to the same reasons. 

This present study has several limitations. In particular, additional apoptosis, cell signaling, and proliferation biomarkers might be assessed, and other techniques, such as Western blot and immunohistochemistry, might prove useful in measuring protein levels and confirming the efficacy of Nar-Dx-NCs against lung cancer. Moreover, further studies are required to assess the naringin and Nar-Dx-NCs dose–response effects on DEN/2AAF-induced lung carcinogenesis and other experimental lung cancer models, such as benzo[a]pyrene-induced lung carcinogenesis in rodents.

## 5. Conclusions

**Figure 8:**
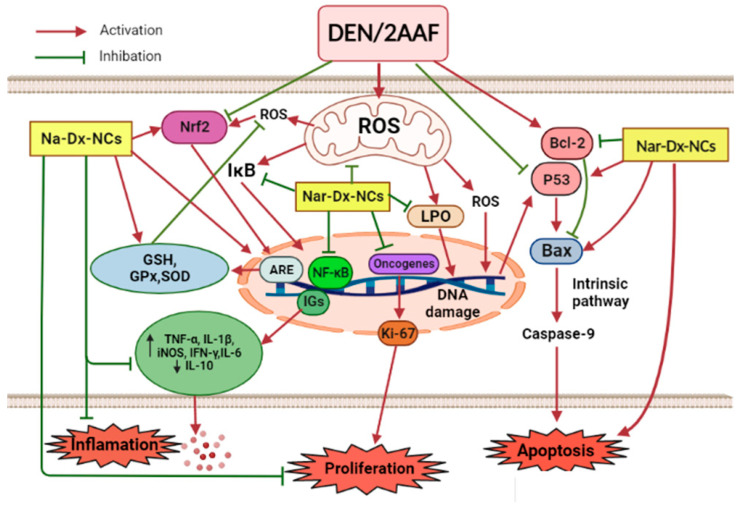
Schematic diagram showing the mechanism of actions of Nar-Dx-NCs against DEN/2AAF-induced lung carcinogenesis, including the suppression of oxidative stress, inflammation, proliferation, and upregulation of apoptosis. Abbreviations: ARE, antioxidant response element; Bax, Bcl-2-associated X protein; Bcl-2, B-cell lymphoma-2; IFN-γ, interferon- γ; IGs, inflammatory genes; IL-6, interleukin-6; IL-10, interleukin-10; iNOS, inducible nitric oxide synthase; NF-κB, nuclear factor-kappa-B; ROS, reactive oxygen species.

The present findings demonstrate that dextrin served as an effective nanocarrier for naringin. This is the first research to document the inhibitory effects of Nar-Dx-NCs on lung cancer. This new formulation diminished lung carcinogenesis induced via DEN/2AAF in different pathways, including the suppression of oxidative stress and inflammation, increasing apoptosis, and decreasing tumor cell proliferation (summarized in Figure 8). Notably, our data reveal Nar-Dx-NCs significantly enhances the anticarcinogenic effects of naringin, thus improving its protective effects against lung cancer. The two important limitations of this study were to assess the effects of Nar-Dx-NCs on normal rats and to compare the anticancer effects of different doses of Nar-Dx-NCs with other existing chemotherapeutic agents. Moreover, although the anticarcinogenic effects of Nar-Dx-NC were confirmed in the lungs of male Wistar rats of this present study, additional preclinical investigations using other animal species and genders, as well as clinical studies, are necessary to confirm the effectiveness of nanocomposites of naringin against human lung cancer.

## Figures and Tables

**Table 1:** Primer sequences for RT-qPCR assays.

Genes	GenBank Accession Number	Sequence (5′–3′)
NF-κB	NM_001276711.1	F: TTCAACATGGCAGACGACGA R: TGCTCTAGTATTTGAAGGTATGGG
Bcl-2	NM_016993.1	F: TAAGCTGTCACAGAGGGGCT R: TGAAGAGTTCCTCCACCACC
Bax	NM_007527.3	F: CTGGATCCAAGACCAGGGTG R: CCTTTCCCCTTCCCCCATTC
P53	NM_030989.3	F: GTTTTTGTTCCTGAGCCCCG R: GAGCAAGGGGTGACTTTGGG
iNOS	NM_012611	F: CTATGGCCGCTTTGATGTGC R: CAACCTTGGTGTTGAAGGCG
IFN-γ	NM_138880.2	F: ACAACCCACAGATCCAGCAC R: CCAGAATCAGCACCGACTCC
IL-6	NM_012589.2	F: CACTTCACAAGTCGGAGGCT R: AGCACACTAGGTTTGCCGAG
IL-10	NM_012854	F: TTGAACCACCCGGCATCTAC R: CCAAGGAGTTGCTCCCGTTA
β-actin	NM_031144.3	F: TCACTATCGGCAATGTGCGG R: GCTCAGGAGGAGCAATGATG

Abbreviations: Bax, Bcl-2-associated X protein; Bcl-2, B-cell lymphoma-2; IFN-γ, interferon-γ; IL-6, interleukin-6; IL-10, interleukin-10; iNOS, inducible nitric oxide synthase; P53 tumor suppressor protein 53; NF-κB, nuclear factor-κB.

**Table 2:** Effect of naringin and Nar-Dx-NCs on lung LPO, GSH content, and GPx and SOD activities in lungs of DEN/2AAF-administered rats.

Groups	LPO (nmole MDA/100 mg Tissue/h)	GSH (nmole /100 mg Tissue)	GPx (mU/100 mg Tissue)	SOD (U/g Tissue)
Normal group	65.06 ± 0.084 ^a^	85.86 ± 1.03 ^d^	214.40 ± 1.40 ^c^	16.76 ± 0.51 ^c^
DEN/2AAF	134.45 ± 0.83 ^c^	44.00 ± 0.75 ^a^	85.61 ± 0.77 ^a^	3.11 ± 0.02 ^a^
DEN/2AAF + Naringin	75.28± 1.06 ^b^	67.30 ± 0.98 ^b^	205.51 ± 0.97 ^b^	9.26 ± 0.12 ^b^
DEN/2AAF + Nar-Dx-NCs	62.73 ± 0.55 ^a^	75.73 ± 0.91 ^c^	212.53 ± 2.52 ^c^	11.03 ± 0.37 ^b^

Data are expressed as mean ± SE (*n* = 6). Means with different superscript symbols (a–d) within the same column are significantly different at *p* < 0.05. Abbreviations: GSH, glutathione; GPx, glutathione peroxidase; LPO, lipid peroxidation; SOD, superoxide dismutase.

## Data Availability

All data are available from corresponding authors upon reasonable request.

## Supplementary Materials

The following supporting information can be downloaded at: https://www.mdpi.com/article/10.3390/cancers15205102/s1, Figure S1: Original Immunoblot Ki-67 replicate 1. Figure S2: Original Immunoblot Ki-67 replicates 2 and 3.

## Abbreviations

2AAF	2-acetylaminofluorene
3-NT	3-nitrotyrosine
ARE	antioxidant response element
Bax	Bcl-2-associated X protein
Bcl-2	B-cell lymphoma-2
CMC	carboxymethyl cellulose
CYP450	cytochrome P450
DEN	diethylnitrosamine
EE	entrapment effectiveness
ELISA	enzyme-linked immunosorbent assay
FTIR	Fourier transformation infrared
GPx	glutathione peroxidase
GSH	glutathione
H&E	hematoxylin and eosin
HRP	horseradish peroxidase
i.p.	Intraperitoneally
IFN-γ	interferon-γ
IGs	inflammatory genes
IL-10	interleukin-10
IL-1β	interleukin-1β
IL-6	interleukin-6
iNOS	inducible nitric oxide synthase
LPO	lipid peroxidation
Nar-Dx-NCs	naringin-dextrin nanocomposite
NF-κB	nuclear factor-κB
NO	nitric oxide
Nrf2	nuclear factor erythroid 2-related factor 2
O2	superoxide anion
ONOO−	peroxynitrite
P53	tumor suppressor protein 53
PCR	polymerase chain reaction
RIPA	radioimmunoprecipitation assay
ROS	reactive oxygen species
RT-qPCR	reverse transcription–quantitative polymerase chain reaction
SDS–PAGE	sodium dodecyl sulfate–polyacrylamide gel electrophoresis
SE	standard error
SOD	superoxide dismutase
TBST	tris-buffered saline with Tween 20
TEM	transmission electron microscopy
TNF-α	tumor necrosis factor-α
UV-Vis	ultraviolet-visible
XRD	X-ray diffraction

## References

[1] Bray F., Ferlay J., Soerjomataram I., Siegel R.L., Torre L.A., Jemal A. (2018). Global cancer statistics 2018: GLOBOCAN estimates of incidence and mortality worldwide for 36 cancers in 185 countries. CA Cancer J. Clin..

[2] Siegel R.L., Miller K.D., Fuchs H.E., Jemal A. (2021). Cancer statistics. CA Cancer J. Clin..

[3] Saber S., Mahmoud A., Helal N., El-Ahwany E., Abdelghany R. (2018). Liver protective effects of renin-angiotensin system inhibition have no survival benefits in hepatocellular carcinoma induced by repetitive administration of diethylnitrosamine in mice. Maced. J. Med. Sci..

[4] Zhang H.E., Henderson J.M., Gorrell M.D. (2019). Animal models for hepatocellular carcinoma. Biochim. Biophy. Acta-Mol. Basis Dis..

[5] Farombi E.O., Shrotriya S., Surh Y.J. (2009). Kolaviron inhibits dimethyl nitrosamine-induced liver injury by suppressing COX-2 and iNOS expression via NF-kappaB and AP-1. Life Sci..

[6] Pradeep K., Raj Mohan C.V., Gobianand K., Karthikeyan S. (2010). Protective effect of *Cassia fistula* Linn. on diethylnitrosamine induced hepatocellular damage and oxidative stress in ethanol pretreated rats. Biol. Res..

[7] Mervai Z., Egedi K., Kovalszky I., Baghy K. (2018). Diethylnitrosamine induces lung adenocarcinoma in FVB/N mouse. BMC Cancer.

[8] Geiger-Maor A., Guedj A., Even-Ram S. (2015). Macrophages regulate the systemic response to DNA damage by a cell nonautonomous mechaism. Cancer Res..

[9] Kishida N., Matsuda S., Itano O., Shinoda M., Kitago M., Yagi H., Abe Y., Hibi T., Masugi Y., Aiura K. (2016). Development of a novel mouse model of hepatocellular carcinoma with nonalcoholic steatohepatitis using a high-fat, choline-defcient diet and intraperitoneal injection of diethylnitrosamine. BMC Gastroenterol..

[10] Downs T.R., Arlt V.M., Barnett B.C., Posgai R., Pfuhler S. (2021). Effect of 2-acetylaminofluorene and its genotoxic metabolites on DNA adduct formation and DNA damage in 3D reconstructed human skin tissue models. Mutagenesis.

[11] Tu X., Ma S., Gao Z., Wang J., Huang S., Chen W. (2017). One-Step Extraction and Hydrolysis of Flavonoid Glycosides in Rape Bee Pollen Based on Soxhlet-Assisted Matrix Solid Phase Dispersion. Phytochem. Anal..

[12] Li H., Zhu F., Chen H., Cheng K.W., Zykova T., Oi N., Lubet R.A., Bode A.M., Wang M., Dong Z. (2014). 6-C-(E-phenylethenyl)-Naringenin Suppresses Colorectal Cancer Growth by Inhibiting Cyclooxygenase-1. Cancer Res..

[13] Liu C., Chu D., Kalantar-Zadeh K., George J., Young H.A., Liu G. (2021). Cytokines: From clinical significance to quantification. Adv. Sci..

[14] Van Gorp H., Lamkanfi M. (2019). The emerging roles of inflammasome-dependent cytokines in cancer development. EMBO Rep..

[15] Quintanilla M., Montero-Montero L., Renart J., Martin-Villar E. (2019). Podoplanin in inflammation and cancer. Int. J. Mol. Sci..

[16] Qian S., Golubnitschaja O., Zhan X. (2019). Chronic inflammation: Key player and biomarker-set to predict and prevent cancer development and progression based on individualized patient profiles. Epma J..

[17] Liu Q., Gao Y., Ci X. (2019). Role of Nrf2 and its activators in respiratory diseases. Oxidative Med. Cell. Longev..

[18] Abouzed T.K., Althobaiti F., Omran A.F., Eldomany E.B., El-Shazly S.A., Alharthi F., Elkattawy A.M., Kahilo K.A., Dorghamm D.A. (2022). The chemoprevention of spirulina platensis and garlic against diethylnitrosamine induced liver cancer in rats via amelioration of inflammatory cytokines expression and oxidative stress. Toxicol. Res..

[19] Sharma P., Kumar V., Guleria P. (2019). Naringin: Biosynthesis and pharmaceutical applications. Indian J. Pharm. Sci..

[20] Ghanbari-Movahed M., Jackson G., Farzaei M.H., Bishayee A.A. (2021). systematic review of the preventive and therapeutic effects of naringin against human malignancies. Front. Pharmacol..

[21] Mintz K.J., Leblanc R.M. (2021). The use of nanotechnology to combat liver cancer: Progress and perspectives. Biochim. Biophys. Acta Rev. Cancer.

[22] Palazzolo S., Bayda S., Hadla M., Caligiuri I., Corona G., Toffoli G., Rizzolio F. (2018). The clinical translation of organic nanomaterials for cancer therapy: A focus on polymeric nanoparticles, micelles, liposomes and exosomes. Curr. Med. Chem..

[23] Yang Z., Shi J., Xie J., Wang Y., Sun J., Liu T., Zhao Y., Zhao X., Wang X., Ma Y. (2020). Large-scale generation of functional mRNA-encapsulating exosomes via cellular nanoporation. Nat. Biomed. Eng..

[24] Wu Z., Ma X., Ma Y., Yang Z., Yuan Y., Liu C. (2020). Core/Shell PEGS/HA hybrid nanoparticle via micelle-coordinated mineralization for tumor-specific therapy. ACS Appl. Mater. Interfaces.

[25] Milewska S., Niemirowicz-Laskowska K., Siemiaszko G., Nowicki P., Wilczewska A.Z., Car H. (2021). Current trends and challenges in pharmacoeconomic aspects of nanocarriers as drug delivery systems for cancer treatment. Int. J. Nanomed..

[26] Ghanbari-Movahed M., Mondal A., Farzaei M.H., Bishayee A. (2022). Quercetin-and rutin-based nano-formulations for cancer treatment: A systematic review of improved efficacy and molecular mechanisms. Phytomedicine.

[27] Ghanbari-Movahed M., Kaceli T., Mondal A., Farzaei M.H., Bishayee A. (2021). Recent advances in improved anticancer efficacies of camptothecin nano-formulations: A systematic review. Biomedicines.

[28] Kashyap D., Tuli H.S., Yerer M.B., Sharma A., Sak K., Srivastava S., Pandey A., Garg V.K., Sethi G., Bishayee A. (2021). Natural product-based nanoformulations for cancer therapy: Opportunities and challenges. Semin. Cancer Biol..

[29] Lagoa R., Silva J., Rodrigues J.R., Bishayee A. (2020). Advances in phytochemical delivery systems for improved anticancer activity. Biotech. Adv..

[30] Mohamed E.E., Abdel-Moneim A., Ahmed O.M., Zoheir K.M., Eldin Z.E., El-Shahawy A.A. (2022). Anticancer activity of a novel naringin‒dextrin nanoformula: Preparation, characterization, and in vitro induction of apoptosis in human hepatocellular carcinoma cells by inducing ROS generation, DNA fragmentation, and cell cycle arrest. J. Drug Deliv. Sci. Technol..

[31] Mohamed E.E., Ahmed O.M., Abdel-Moneim A., Zoheir K.M., Elesawy B.H., Al Askary A., El-Shahawy A.A. (2022). Protective Effects of Naringin–Dextrin Nanoformula against Chemically Induced Hepatocellular Carcinoma in Wistar Rats: Roles of Oxidative Stress, Inflammation, Cell Apoptosis, and Proliferation. Pharmaceuticals.

[32] Manchun S., Dass C.R., Sriamornsak P. (2014). Designing nanoemulsion templates for fabrication of dextrin nanoparticles via emulsion cross-linking technique. Carbohydr. Polym..

[33] Morsy H.M., Ahmed O.M., Zoheir K.M.A., Abdel-Moneim A. (2023). The anticarcinogenic effect of eugenol on lung cancer induced by diethylnitrosamine/2-acetylaminofluorene in Wistar rats: Insight on the mechanisms of action. Apoptosis.

[34] Abdel-Moneim A., Ahmed O.M., El-Twab A., Sanaa M., Zaky M.Y., Bakry L.N. (2021). Prophylactic effects of *Cynara scolymus* L. leaf and flower hydroethanolic extracts against diethylnitrosamine/acetylaminoflourene-induced lung cancer in Wistar rats. Environ. Sci. Pollut. Res..

[35] Camargo C.A., Gomes-Marcondes M.C., Wutzki N.C., Aoyama H. (2012). Naringin inhibits tumor growth and reduces interleukin-6 and tumor necrosis factor α levels in rats with Walker 256 carcinosarcoma. Anticancer Res..

[36] Bancroft J.D., Gamble M. (2008). Theory and Practice of Histological Techniques.

[37] Abràmoff M.D., Magalhes P.J., Ram S.J. (2004). Image processing with imageJ. Biophoton Int..

[38] Sthoeger Z., Zinger H., Sharabi A., Asher I., Mozes E. (2013). The tolerogenic peptide, hCDR1, down-regulates the expression of interferon-α in murine and human systemic lupus erythematosus. PLoS ONE.

[39] Sivalingam K., Amirthalingam V., Ganasan K., Huang C.Y., Viswanadha V.P. (2019). Neferine suppresses diethylnitrosamine-induced lung carcinogenesis in Wistar rats. Food Chem. Toxicol..

[40] Stabrauskiene J., Kopustinskiene D.M., Lazauskas R., Bernatoniene J. (2022). Naringin and naringenin: Their mechanisms of action and the potential anticancer activities. Biomedicines.

[41] Ahmed O.M., Fahim H.I., Mohamed E.E., Abdel-Moneim A. (2022). Protective effects of *Persea americana* fruit and seed extracts against chemically induced liver cancer in rats by enhancing their antioxidant, anti-inflammatory, and apoptotic activities. Environ. Sci. Pollut. Res..

[42] Barrera G. (2012). Oxidative stress and lipid peroxidation products in cancer progression and therapy. Int. Sch. Res. Not..

[43] Kim J.K., Park J.H., Ku H.J., Kim S.H., Lim Y.J., Park J.W., Lee J.H. (2018). Naringin protects acrolein-induced pulmonary injuries through modulating apoptotic signaling and inflammation signaling pathways in mice. J. Nutr. Biochem..

[44] Akintunde J.K., Abioye J.B., Ebinama O.N. (2020). Potential protective effects of naringin on oculo-pulmonary injury induced by PM10 (wood smoke) exposure by modulation of oxidative damage and acetylcholine esterase activity in a rat model. Curr. Ther. Res. Clin. Exp..

[45] Cavia-Saiz M., Busto M.D., Pilar-Izquierdo M.C., Ortega N., Perez-Mateos M., Muniz P. (2010). Antioxidant properties, radical scavenging activity and biomolecule protection capacity of flavonoid naringenin and its glycoside naringin: A comparative study. J. Sci. Food Agric..

[46] Askari V.R., Shafiee-Nick R. (2018). Promising neuroprotective effects of β-caryophyllene against LPS-induced oligodendrocyte toxicity: A mechanistic study. Biochem. Pharmacol..

[47] Jawa R.S., Anillo S., Huntoon K., Baumann H., Kulaylat M. (2011). Interleukin-6 in surgery, trauma, and critical care part II: Clinical implications. J. Intensive Care Med..

[48] Janakiram N.B., Valerio M.S., Goldman S.M., Dearth C.L. (2021). The role of the inflammatory response in mediating functional recovery following composite tissue injuries. Int. J. Mol. Sci..

[49] Man S., Li J., Qiu P., Liu J., Liu Z., Ma L., Gao W. (2017). Inhibition of lung cancer in diethylnitrosamine-induced mice by *Rhizoma paridis* saponins. Mol. Carcinog..

[50] Wu Y., Sreeharsha N., Sharma S., Mishra A., Singh A.K., Gubbiyappa S.K. (2020). Anticancer effect of rosiglitazone, a PPAR-γ agonist against Diethylnitrosamine-induced lung carcinogenesis. ACS Omega.

[51] Cicek B., Hayme S., Kuzucu M., Cetin A., Yeni Y., Genc S., Yildirim S., Bolat I., Kantarci M., Gul M. (2022). Sorafenib Alleviates Inflammatory Signaling of Tumor Microenvironment in Lung Cancer. Res. Sq..

[52] Lin X., Ju Y.N., Gao W., Li D.M., Guo C.C. (2018). Desflurane attenuates ventilator-induced lung injury in rats with acute respiratory distress syndrome. BioMed. Res. Int..

[53] Liu W., Liu K., Zhang S., Shan L., Tang J. (2018). Tetramethylpyrazine showed therapeutic effects on sepsis-induced acute lung injury in rats by inhibiting endoplasmic reticulum stress protein kinase RNA-like endoplasmic reticulum kinase (PERK) signaling-induced apoptosis of pulmonary microvascular endothelial cells. Med. Sci. Monit. Int. Med. J. Exp. Clin. Res..

[54] Yang Y., Huang Z., Li J., Mo Z., Huang Y., Ma C., Wang W., Pan X., Wu C. (2019). PLGA porous microspheres dry powders for codelivery of afatinib-loaded solid lipid nanoparticles and paclitaxel: Novel therapy for EGFR tyrosine kinase inhibitors resistant nonsmall cell lung cancer. Adv. Healthc. Mater..

[55] Aggarwal B.B., Shishodia S., Sandur S.K., Pandey M.K., Sethi G. (2006). Inflammation and cancer: How hot is the link?. Biochem. Pharmacol..

[56] Rojo de la Vega M., Chapman E., Zhang D.D. (2018). NRF2 and the Hallmarks of Cancer. Cancer Cell.

[57] Sánchez-Ortega M., Carrera A.C., Garrido A. (2021). Role of NRF2 in lung cancer. Cells.

[58] Hamzavi M., Tadbir A.A., Rezvani G., Ashraf M.J., Fattahi M.J., Khademi B., Sardari Y., Jeirudi N. (2013). Tissue expression, serum and salivary levels of IL-10 in patients with head and neck squamous cell carcinoma. Asian Pac. J. Cancer Prev..

[59] Kobelt D., Zhang C., Clayton-Lucey I.A., Glauben R., Voss C., Siegmund B., Stein U. (2020). Pro-inflammatory TNF-α and IFN-γ Promote Tumor Growth and Metastasis via Induction of MACC1. Front. Immunol..

[60] Jorgovanovic D., Song M., Wang L., Zhang Y. (2020). Roles of IFN-γ in tumor progression and regression: A review. Biomark. Res..

[61] Todoric J., Antonucci L., Karin M. (2016). Targeting Inflammation in Cancer Prevention and Therapy. Cancer Prev. Res..

[62] Ginwala R., Bhavsar R., Chigbu D.G., Jain P., Khan Z.K. (2019). Potential role of flavonoids in treating chronic inflammatory diseases with a special focus on the anti-inflammatory activity of apigenin. Antioxidants.

[63] Hamza A.A., Heeba G.H., Elwy H.M., Murali C., El-Awady R., Amin A. (2018). Molecular characterization of the grape seeds extract’s effect against chemically induced liver cancer: In vivo and in vitro analyses. Sci. Rep..

[64] Rashid S., Ali N., Nafees S., Ahmad T., Hasan S.K., Sultana S. (2013). Abrogation of 5-flourouracil induced renal toxicity by bee propolis via targeting oxidative stress and inflammation in Wistar rats. J. Pharm. Res..

[65] Zhao Y., Xu Y., Li Y., Xu W., Luo F., Wang B., Pang Y., Xiang Q., Zhou J., Wang X. (2013). NF-κB-mediated inflammation leading to EMT via miR-200c is involved in cell transformation induced by cigarette smoke extract. Toxicol. Sci..

[66] Atiq A., Shal B., Naveed M., Khan A., Ali J., Zeeshan S., Al-Sharari S.D., Kim Y.S., Khan S. (2019). Diadzein ameliorates 5-fluorouracil-induced intestinal mucositis by suppressing oxidative stress and inflammatory mediators in rodents. Eur. J. Pharmacol..

[67] Hayaza S., Darmanto W., Wahyuningsih S.P.A., Susilo R.J.K., Husen S.A., Winarni D., Doong R.A. (2022). Immunomodulatory activity of Okra raw polysaccharide extract by regulating TNF-A, IFN-G Levels, and cell apoptosis in DEN-induced mice. Res. J. Pharm. Technol..

[68] Zhang H.W., Hu J.J., Fu R.Q., Liu X., Zhang Y.H., Li J., Liu L., Li Y.N., Deng Q., Luo Q.S. (2018). Flavonoids inhibit cell proliferation and induce apoptosis and autophagy through downregulation of PI3Kγ mediated PI3K/AKT/mTOR/p70S6K/ULK signaling pathway in human breast cancer cells. Sci. Rep..

[69] Unsal V., Kurutaş E.B. (2017). Experimental Hepatic Carcinogenesis: Oxidative Stress and Natural Antioxidants. Maced. J. Med. Sci..

[70] Pacher P., Beckman J.S., Liaudet L. (2007). Nitric oxide and peroxynitrite in health and disease. Physiol. Rev..

[71] Bishayee A., Barnes K.F., Bhatia D., Darvesh A.S., Carroll R.T. (2010). Resveratrol suppresses oxidative stress and inflammatory response in diethylnitrosamine-initiated rat hepatocarcinogenesis. Cancer Prev. Res..

[72] Yu X., Ge L., Niu L., Lian X., Ma H., Pang L. (2018). The dual role of inducible nitric oxide synthase in myocardial ischemia/reperfusion injury: Friend or foe?. Oxidative Med. Cell. Longev..

[73] Ahmad S.F., Attia S.M., Bakheet S.A., Zoheir K.M., Ansari M.A., Korashy H.M., Abdel-Hamied H.E., Ashour A.E., Abd-Allah A.R. (2015). Naringin attenuates the development of carrageenan-induced acute lung inflammation through inhibition of NF-κb, STAT3 and pro-inflammatory mediators and enhancement of IκBα and anti-inflammatory cytokines. Inflammation.

[74] Liu Y., Wu H., Nie Y.C., Chen J.L., Su W.W., Li P.B. (2011). Naringin attenuates acute lung injury in LPS-treated mice by inhibiting NF-κB pathway. Int. Immunopharmacol..

[75] Habauzit V., Sacco S.M., Gil-Izquierdo A., Trzeciakiewicz A., Morand C., Barron D., Pinaud S., Offord E., Horcajada M.N. (2011). Differential effects of two citrus flavanones on bone quality in senescent male rats in relation to their bioavailability and metabolism. Bone.

[76] Chtourou Y., Aouey B., Kebieche M., Fetoui H. (2015). Protective role of naringin against cisplatin induced oxidative stress, inflammatory response and apoptosis in rat striatum via suppressing ROS-mediated NF-κB and P53 signaling pathways. Chem. Biol. Interact..

[77] Lv Z., Wu W., Ge S., Jia R., Lin T., Yuan Y., Kuang H., Yang B., Wu L., Wei J. (2018). Naringin protects against perfluorooctane sulfonate-induced liver injury by modulating NRF2 and NF-κB in mice. Int. Immunopharmacol..

[78] Purvis J.E., Karhohs K.W., Mock C., Batchelor E., Loewer A., Lahav G. (2012). p53 dynamics control cell fate. Science.

[79] Ou X., Lu Y., Liao L., Li D., Liu L., Liu H., Xu H. (2015). Nitidine chloride induces apoptosis in human hepatocellular carcinoma cells through a pathway involving p53, p21, Bax and Bcl-2. Oncol. Rep..

[80] Campbell K.J., Tait S.W. (2018). Targeting BCL-2 regulated apoptosis in cancer. Open Biol..

[81] Kang M.H., Reynolds C.P. (2009). Bcl-2 inhibitors: Targeting mitochondrial apoptotic pathways in cancer therapy. Clin. Cancer Res..

[82] Zhu L., Han M.B., Gao Y., Wang H., Dai L., Wen Y., Na L.X. (2015). Curcumin triggers apoptosis via upregulation of Bax/Bcl-2 ratio and caspase activation in SW872 human adipocytes. Mol. Med. Rep..

[83] Liu Z., Ding Y., Ye N., Wild C., Chen H., Zhou J. (2016). Direct activation of Bax protein for cancer therapy. Med. Res. Rev..

[84] Liu X., Fan L., Lu C., Yin S., Hu H. (2020). Functional role of p53 in the regulation of chemical-induced oxidative stress. Oxidative Med. Cell. Longev..

[85] Qi F., Li A., Inagaki Y. (2012). Induction of apoptosis by cinobufacini preparation through mitochondria- and Fas-mediated caspase-dependent pathways in human hepatocellular carcinoma cells. Food Chem. Toxicol..

[86] Chen R., Qi Q.L., Wang M.T., Li Q.Y. (2016). Therapeutic potential of naringin: An overview. Pharm. Biol..

[87] Abotaleb M., Samuel S.M., Varghese E., Varghese S., Kubatka P., Liskova A., Büsselberg D. (2018). Flavonoids in cancer and apoptosis. Cancers.

[88] Raina R., Hussain A., Sharma R. (2020). Molecular insight into apoptosis mediated by flavones in cancer. World Acad. Sci. J..

[89] Tramm T., Kyndi M., Sørensen F.B., Overgaard J., Alsner J. (2018). Influence of intra-tumoral heterogeneity on the evaluation of BCL2, E-cadherin, EGFR, EMMPRIN, and Ki-67 expression in tissue microarrays from breast cancer. Acta Oncol..

[90] Wen S., Zhou W., Li C.M., Hu J., Hu X.M., Chen P., Shao G.L., Guo W.H. (2015). Ki-67 as a prognostic marker in early-stage non-small cell lung cancer in Asian patients: A meta-analysis of published studies involving 32 studies. BMC Cancer.

[91] Wang D., Ye W., Shi Q. (2021). Prognostic Value of Ki-67 Expression in Advanced Lung Squamous Cell Carcinoma Patients Treated with Chemotherapy. Cancer Manag. Res..

[92] De Jong W.H., Borm P.J. (2008). Drug delivery and nanoparticles: Applications and hazards. Int. J. Nanomed..

