# Supplementary figures and images for: Naringin–Dextrin Nanocomposite Abates Diethylnitrosamine/Acetylaminofluorene-Induced Lung Carcinogenesis by Modulating Oxidative Stress, Inflammation, Apoptosis, and Cell Proliferation
Source: Cancers (Basel). 2023 Oct 22;15(20):5102. doi: 10.3390/cancers15205102 (PMC10605195; doi:10.3390/cancers15205102)

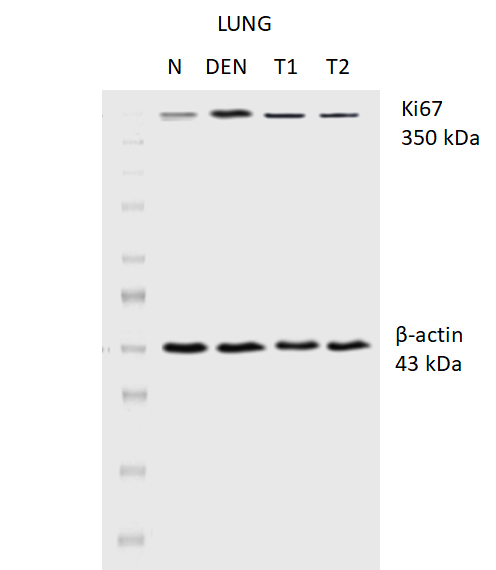

Supplement: Supplementary file 1 [file cancers-15-05102-s001.zip › Figure S1 Original Immunoblot Ki-67 replicate 1.jpg]

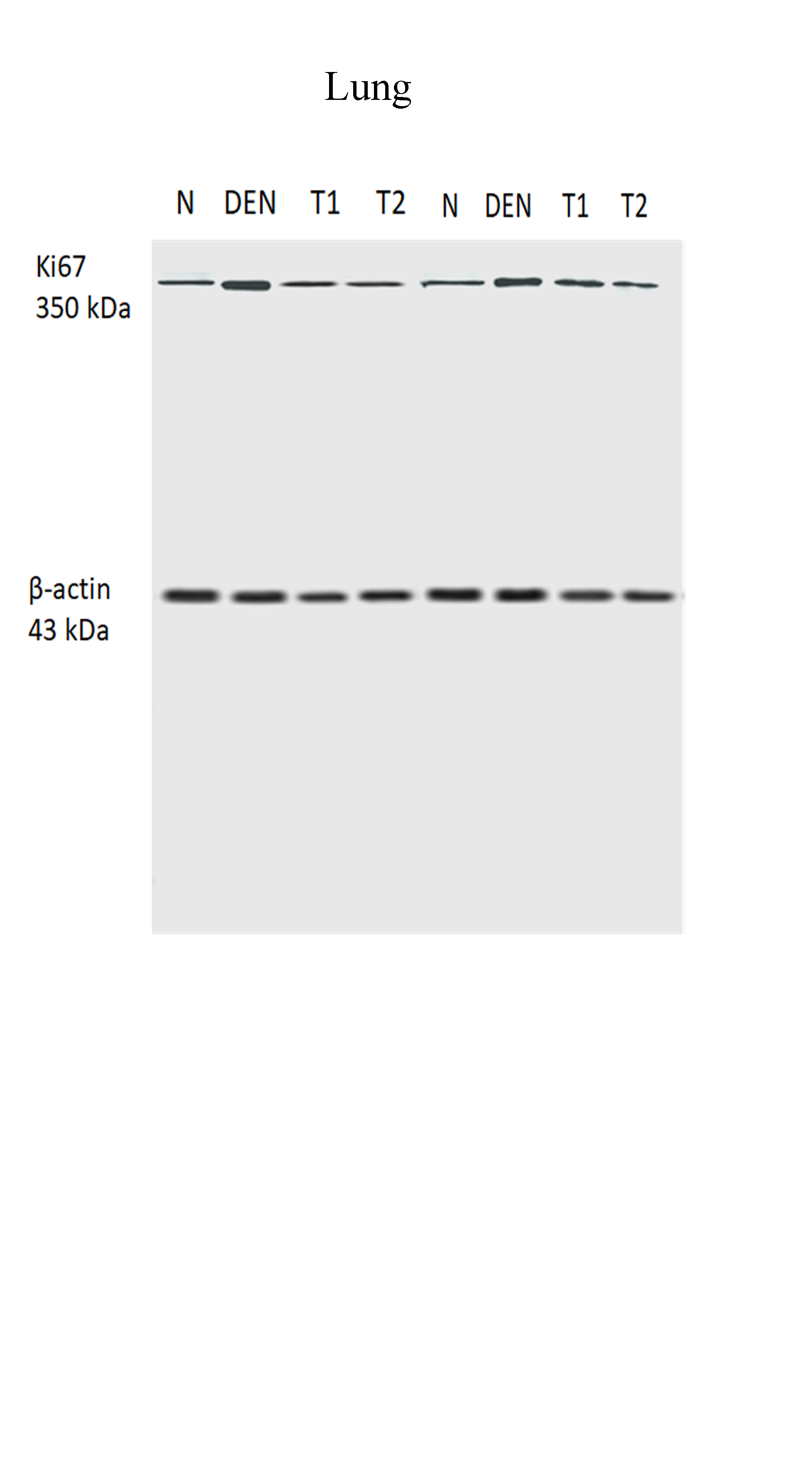

Supplement: Supplementary file 1 [file cancers-15-05102-s001.zip › Figure S2 Original Immunoblot Ki-67 replicates 2 and 3.jpg]
